# Developing Targeted Health Service Interventions Using the PRECEDE-PROCEED Model: Two Australian Case Studies

**DOI:** 10.1155/2012/279431

**Published:** 2012-07-17

**Authors:** Jane L. Phillips, John X. Rolley, Patricia M. Davidson

**Affiliations:** ^1^School of Nursing, The University of Notre Dame Australia, The Cunningham Centre for Palliative Care, St Vincent's & Mater Health Sydney, 170 Darlinghurst Road, Sydney, NSW 2010, Australia; ^2^Cardiac Investigation Unit, St Vincent's Hospital, P.O. Box 2900, Fitzroy, VIC 3065, Australia; ^3^Cardiovascular Nursing Research, St Vincent's Hospital and Centre for Cardiovascular and Chronic Care, Faculty of Nursing, Midwifery & Health, University of Technology Sydney, Broadway, NSW 2007, Australia

## Abstract

*Aims and Objectives*. This paper provides an overview of the applicability of the PRECEDE-PROCEED Model to the development of targeted nursing led chronic illness interventions. *Background*. Changing health care practice is a complex and dynamic process that requires consideration of social, political, economic, and organisational factors. An understanding of the characteristics of the target population, health professionals, and organizations plus identification of the determinants for change are also required. Synthesizing this data to guide the development of an effective intervention is a challenging process. The PRECEDE-PROCEED Model has been used in global health care settings to guide the identification, planning, implementation, and evaluation of various health improvement initiatives. *Design*. Using a reflective case study approach, this paper examines the applicability of the PRECEDE-PROCEED Model to the development of targeted chronic care improvement interventions for two distinct Australian populations: a rapidly expanding and aging rural population with unmet palliative care needs and a disadvantaged urban community at higher risk of cardiovascular disease. *Results*. The PRECEDE-PROCEED Model approach demonstrated utility across diverse health settings in a systematic planning process. In environments characterized by increasing health care needs, limited resources, and growing community expectations, adopting planning tools such as PRECEDE-PROCEED Model at a local level can facilitate the development of the most effective interventions. *Relevance to Clinical Practice*. The PRECEDE-PROCEED Model is a strong theoretical model that guides the development of realistic nursing led interventions with the best chance of being successful in existing health care environments.

## 1. Introduction

Globally, over the past decade there has been increasing recognition of the need for effective management of chronic and complex conditions and less dependence on acute care services [1, 2]. Achieving this magnitude of reform has been difficult because it requires reorientation of health services, a greater focus on primary health care, and an enduring commitment to the delivery of best evidence-based practice. This increased emphasis on evidence-based practice dictates that a systematic and critical analysis of priorities and presumed causes be undertaken to guide health service planning [1, 3]. Yet, health professionals frequently rely solely on intuition or anecdotal information to identify or address a particular health problem at a population level as opposed to empirical research [4].

### 1.1. Needs Assessment

A Needs Assessment is a complex, multidimensional process which provides information and evidence to inform the objective and valid tailoring of health services or commissioning of new initiatives. The needs assessment process ensures due consideration is given to the quality of the evidence relevant to the risks and benefits of specific interventions [5]. Identifying the priority health problem and analysing the problem is often the catalyst that enables services to reorientate care delivery from being institutionally focused to addressing populations needs [6]. This systematic process facilitates appraisal of a population's health needs, identifies service gaps, the services required and the degree to which the proposed service(s) will be used by those in greatest need [7]. All relevant information, concerning health-related needs, and possible solutions to enable the planning and delivery of cost-effective services or new initiatives are collated [8]. This data enables navigation of a pathway forward, while balancing the clinical, ethical, and economic consideration of “need” [9]. Identifying a number of worthy needs can make determining the health priority the most difficult stage of the needs assessment, particularly as limited resources necessitate prioritisation. Despite various criteria having been put forward to assist prioritising health problems, the absence of an evaluation formula requires decision makers to subjectively determine where to direct health-care resources [10]. The PRECEDE-PROCEED Model endeavors to addresses this limitation.

## 2. Aim

The purpose of this paper is to demonstrate the applicability of the PRECEDE-PROCEED Model (“Model”) to the development of specific chronic care interventions for two distinct Australian populations: a rapidly growing and ageing rural population with unmet palliative care needs (R-PAC Project) and an urban community at higher risk of cardiovascular disease (APRICA 2 Project). The achievement of a comprehensive understanding of the health problem in each population, stakeholder engagement, and the development of tailored interventions signaled the completion of the PRECEDE phases of the process, which is the focus of this paper.

## 3. Methods

### 3.1. Applying the PRECEDE-PROCEED Model

This conceptual model minimizes the risk of subjectivity by synthesizing disparate sources of data to ensure that initiatives with the greatest potential of achieving the best health outcomes are implemented [10]. The Model is based on the premise that the determinants of health and health risks are multi-factorial and that multifaceted and multisectoral efforts are required to effect behavioural, environmental, and social change [11].

The Model has evolved from a diagnostic tool developed in the 1980s, into a nine-phase model that integrates environmental health factors and evaluation into the process [10]. PRECEDE is an acronym that stands for predisposing, reinforcing, and enabling constructs in education, diagnosis, and evaluation, while PROCEED is the second part of the conceptual model and involves four phases that are focused on implementation and evaluation [10]. These processes work in unison with the PRECEDE phases facilitating the identification of priorities and the setting of objectives, while the PROCEDE phases assist in identifying the criteria for policy implementation and subsequent evaluation [10].

A major strength of this Model is its capacity to facilitate identification of the desired outcomes at the outset of the planning process, which determines the evaluation metrics [10]. This Model also aids systematic classification of factors by their relative importance and capacity for modification through the use of a ranking system [10]. A ranking system facilitates consideration of the determinants for change at individual, provider, and system levels and allows for the identification, development, and implementation of interventions with the greatest potential of achieving a positive impact. Over the past two decades, the Model has been used internationally by health care planners and researchers to design interventions that acknowledge a wide range of individual and environmental determinants of health [12–15].

### 3.2. Setting

**R-PAC Project**

This initiative was undertaken as one component of a larger project, which aimed to strengthen partnerships to improve the coordination and delivery of local palliative care services [16]. This project was undertaken in a regional Australian coastal community, with a population of 67 0000. Over the past 20 years this community has experienced unprecedented population grown due to the internal migration of retirees [17]. It is anticipated that the areas popularity as a preferred retirement destination for baby boomers will continue, with 35% of population growth expected to be in the over 65 age group by 2031 [18]. By this time, estimates suggest that this area will have the highest proportion of over 65-year olds in the state [18].

**APRICA 2 Project**

This practice improvement intervention was undertaken in the Greater Western Sydney region, which is characterised by a diverse population who experience greater educational disadvantage, lower-levels of employment and employment capacity, and below-average annual income [19]. These determinants all impact adversely on how people perceive their risk for cardiovascular disease (CVD) [20]. The area also has higher proportions of non-English speaking residents [21], with some culturally and linguistically diverse groups being at a potentially greater risk of CVD [22].

The geographical spread of this outer urban community combined with the higher prevalence of socioeconomic disadvantage also adversely impacts on the populations' access to healthcare and appropriate transport [19].

### 3.3. Governance

The formation of critical reference groups comprising key stakeholders as part of the governance of the R-PAC and APRICA 2 Projects reflects the Models emphasis on assessing the social determinants of the population and engaging community stakeholders. Active stakeholder input ensured that the problems and priorities were defined by the community as opposed to being imposed by external parties.

### 3.4. Data Sources

Multiple sources of data were considered during the projects, including a comprehensive review of the literature and local policy documents and content emerging from key informant interviews. The Model facilitated the synthesis of the social assessment data which enabled a link between the priority health problems and the communities' needs to be established and focused the planning process.

### 3.5. Deriving Outcomes

In each project the desired outcomes were identified and defined at the outset of the planning process, which facilitated the development of specific and measurable evaluation metrics at the process (Phase 7), impact (Phase 8), and outcome (Phase 9) evaluation levels. The Model aided systematic classification of factors by their relative importance and capacity for modification for both projects [10, 23]. This ranking system facilitated consideration of the determinants for change at individual, provider, and system levels and allowed for the identification, development, and implementation of tailored interventions with the greatest potential of achieving a positive impact. Evidence suggests that improvement strategies that attend to the highest ranked predisposing, enabling, and reinforcing factors are those most likely to be successful [10, 24, 25]. Adopting this Model to plan health service improvement helps to optimise the use of scarce health resources (time, personnel, services, finances) by developing interventions that are likely to have the most impact, based on importance and changeability [10].

## 4. Applying PRECEDE-PROCEED Model: Two Case Studies

The Model calls for a deductive approach to assessing populations unmet needs. The complexities associated with the impacts on quality of life, health, behavior, the environment, and factors associated with achieving a desired outcome (predisposing, enabling, and reinforcing factors) for the populations identified as having unmet needs in the two case studies presented in this paper; and how identified health priorities guided subsequent intervention development and evaluation. A summary of the Model's phases as they relate of the R-PAC and APRICIA 2 Projects, are summarized consecutively in the sections below as case studies.

### 4.1. Phases 1 and 2: Social Assessment and Epidemiological Assessment

Addressing a population unmet needs and improving their quality of life is the Models' aspiration goal. Identifying and evaluating the various social problem(s) which impact on the quality of life of the target populations made undertaking a “social and epidemiological assessment” an important first step towards achieving this goal.

**R-PAC Project**

**Figure 1:**
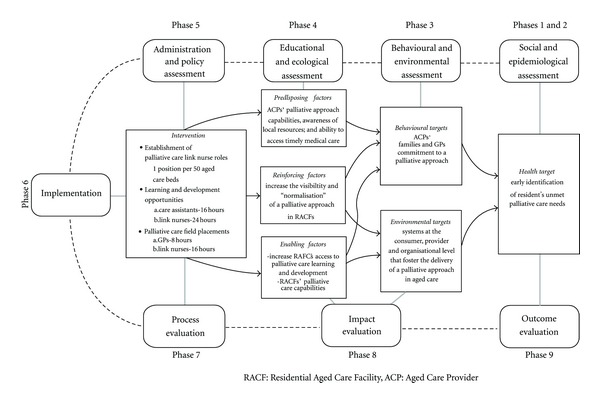
Overview of the PRECEDE-PROCEED Model as applied to the R-PAC Project.

To assist with the systematic identification of local palliative care priorities, a focussed needs' assessment was undertaken at the outset of the project [17]. Synthesis of this data established a link between the priority health problems and the communities' needs and identified that improving the delivery of evidence-based palliative care was a key community priority (Figure 1) [17].

**APRICA 2 Project**

Similarly, the APRICA 2 Projects' needs assessment revealed a need to improve secondary CVD prevention after percutaneous coronary interventions (PCIs) for a diverse urban population in the outer fringe of Western Sydney. The social determinates of educational disadvantage, underemployment, employment capacity, and below-average annual income have all been identified as impacting adversely on this populations perceived CVD risk [26]. The higher proportions of cultural groups at increased risk of CVD [20], combined with more limited transport and healthcare resources, made focusing on reducing this disadvantaged populations secondary CVD risks a priority for the APRICA 2 Project [21].

### 4.2. Phase 3: Behavioural and Environmental Assessment

The “behavioural and environmental assessment” facilitated identification of the specific health problems that may contribute to the target populations' quality of life, social goals or problems [10]. This phase assisted in identifying risk factors that deserve priority based on their perceived importance and changeability [10].

**R-PAC Project**

The evidence that emerged from Phase 1 and 2 suggested that the inward migration of retirees to this regional community was projected to continue and the burden of progressive life limiting diseases would increase in line with population aging, impacting adversely on the capacity of the existing palliative care service to meet growing demand [17]. Many older people requiring palliative care are admitted to the acute or nursing home setting as a result of caregiver burden, living alone and/or their care needs exceed available community services [17]. End-of-life care in nursing homes is provided by nonspecialist providers, for whom care of the dying is not their primary focus making building workforce palliative care capacity a priority [27]. The behavioural issues impacting on the delivery of palliative care to older people exposed a need to increase palliative care access to people with a progressive nonmalignant life limiting illnesses and to enhance palliative care delivery in local nursing homes. Addressing the palliative care needs of older people in aged care was strongly aligned with a national agenda and the release of evidence-based guidelines [27]. Given the availability of funding to strengthen local palliative care partnerships [16], it was considered that positive changes could be achieved during the project period.

**APRICA 2 Project**

The Phases 1 and 2 data revealed that the area had a higher than state average acute coronary-related admission and readmission rates [22], with people born overseas, who are overweight or obese and smokers being overrepresented [19]. Factors such as smoking, obesity, inactivity, and low uptake and completion rates of secondary prevention programs such as cardiac rehabilitation are all known to contribute to short- and long-term CVD mortality and morbidity [28]. Health professional behaviours inadvertently increasing the population's secondary CVD risks were identified during this process, including poor adherence to evidence-based guidelines and limited followup and promotion of cardiac rehabilitation programs to post, PCI patients. Compounding the populations' secondary CVD risks were environmental factors such as limited access to appropriate secondary CVD resources, participation in cardiac rehabilitation programs, carer engagement in healthcare decision-making, and secondary prevention activities in the acute care setting.

At the completion of Phase 3, the health priorities for each project were evident, which allowed for the establishment of project objectives, with clearly defined target populations (WHO), desired outcomes (WHAT), and degree to which the target population will benefit (HOW MUCH) within a specific period (WHEN).

### 4.3. Phase 4: Educational and Ecological Assessment

An “educational and ecological assessment” facilitates categorising the predisposing, enabling or reinforcing factors contributing to the behaviours previously identified [10]. This phase facilitates systematic identification of health problems and associated risk factors that deserve priority based on their perceived importance and changeability, whilst considering the effective allocation of limited resources [10]. Importantly, this stage focuses on the development of the intervention to address the identified health problem. Having the critical reference groups assess and ranked the predisposing, reinforcing or enabling factors helps drive the change management processes [29, 30].

### 4.4. Predisposing Factors

**R-PAC Project**

Predisposing factors ranked highest as acting to either motivate or inhibit the delivery of a palliative approach in local nursing homes, included aged care personnels palliative care awareness, knowledge, competencies, and confidence; access to the specialist palliative care for residents with complex palliative care needs; the number of general practitioners (GPs) prepared to review residents in local nursing homes; residents' and families' awareness of a palliative approach and involvement in care planning [31].

**APRICA 2 Project**

The highest ranking predisposing factors for the APRIC 2 population were identified as being a pervading sense of being “cured” following PCI [32], inadequate understanding of the need for secondary prevention following PCI, wide diversity in PCI nursing care practices across institutions; inadequate communication between acute and primary care providers, low referral rates to secondary prevention programs, and poor uptake and completion of secondary CVD prevention programs by patients undergoing PCIs.

### 4.5. Reinforcing Factors

**R-PAC Project**

In the aged care setting, residents, family members, other health care providers, peers, and educators play a role in reinforcing positive and negative behaviours through rewards, feedback, and punishments [10]. The reinforcing factors considered most important and amenable to change included the need to increase age care personnel's awareness of the specialist palliative care referral process, develop appropriate systems for GPs to be routinely engaged in resident's end-of-life care planning, provide residents and families with information about a palliative approach, and increase the visibility and “normalisation” of a palliative approach in aged care [29].

**APRICA 2 Project**

The reinforcing factors ranked highest in terms of importance and changeability included the lack of national, state, or local PCI evidence-based nursing care guidelines; no linkage between PCI nursing care delivery in acute care and secondary CVD prevention programs; patients' limited participation in secondary CVD prevention programs.

### 4.6. Enabling Factors

**R-PAC Project**

Whereas enabling factors such as: accessibility, availability and skills impacted on the aged care personnel's ability to deliver a palliative approach were the most highly ranked factors. Further analysis revealed that these enabling factors included aged care personnel's capacity to: effectively communicate clinical findings to external health professionals; effectively advocate on behalf of the residents; utilise a common palliative care language, both within aged care and with external health professionals; arrange timely access to palliative care equipment; refer the resident to a specialist palliative care team; acquire greater palliative care competencies and confidence; and access palliative care education opportunities locally [29]. A range of enabling factors were also acting to limit residents' access to palliative care as a result of: a lower ratio of registered nurses as a proportion of the total aged care workforce; aged care personnels limited palliative care knowledge, skills and confidence; under utilisation of the specialist palliative care team; difficulty accessing timely and appropriate GP input, specialist support, medications and equipment; and residents' and families' limited awareness and understanding of a palliative approach [29, 30]. Acknowledging the availability and accessibility of resources along with the competencies required to implement the intervention ensures that the highest ranked factors, in terms of importance and changeability become the focus of the intervention [10].

**APRICA 2 Project**

Health professionals' willingness to engage in and lead CVD quality improvement activities; the encouragement and support provided by families/carers to enable the patient to reduce their CVD risk; and capacity of the peak cardiovascular organisations to promote a national PCI quality improvement agenda, were identified as being critical enabling factors for change. Integrating relevant data into the Model enabled a comprehensive picture of the cardiovascular health needs of an urban population to be identified and guided identification of the action required, including: development national PCI evidence-based nursing guidelines integrating secondary prevention [32, 33]; increasing uptake of cardiac rehabilitation post PCIs; informing patients and carers of available social support(s), reinforcing the importance of secondary prevention and details on accessing local CVD secondary prevention information and programs. As risk modification is dependent upon the individual's perception of risk, identifying and ranking these factors was critical to shaping the APRICA Project intervention, in Phase 4 [33].

### 4.7. Phase 5: Administrative and Policy Assessment

**R-PAC Project**

The data shaped the development of a multifaceted intervention development focused on increasing aged care personnel's palliative care capacity [34]. The unique learning needs of the various categories of personnel delivering care in the aged care setting dictated the development of tailored learning strategies reflecting their scope of practice [34]. An assessment of the organisational and administrative capabilities allowed for identification of the resources required for the development and implementation of the proposed intervention and consideration of factors that may hinder the proposed change [10]. This process confirmed that the nine participating nursing homes' organisational missions were compatible with the projects goals and the planned intervention objectives [10]. Administrative and policy issues that needed to be factored into the intervention development, including time constraints, change management and the need for a “clinical champion” in each nursing home. Consideration of other dynamics, such as staff shortages, accreditation schedules, commissioning of new beds, and the execution of other initiatives helped determine the extent and speed by which the proposed intervention could be implemented.

**APRICA 2 Project**

Acute care workforce shortages, frequent patient transfers across institutions, financial pressures, and the need for increased efficiency all had the potential to adversely impact on the implementation of any intervention developed to improve secondary CVD risk after PCI [35]. These complex administrative and policy conditions made the recruitment of local “change champions” to facilitate guideline and intervention implementation and actively engaging Australia and New Zealand's peak cardiovascular nursing organisations in PCI guideline development, important strategies to address these barriers. This reality guided the developed of the subobjectives developed to address these priorities.

### 4.8. Phase 6: Implementation of the Intervention

Implementation of the intervention (Phase 6) marks the commencement of the PROCEED component of the Model.

**R-PAC Project**

The multifaceted intervention implemented sought to work with aged care personnel to increase resident's access to palliative care by creating an enabling and empowering learning environment. The strategies employed included increasing access to specialists' resources and evidence-based information through instituting: a clinical champion “link nurse” role, increasing learning and development opportunities for nurses, care assistants, and GPs, and promoting networking and multidisciplinary care planning processes [34].

**APRICA 2 Project**

The study has completed the development of a set of evidence-based guidelines for the care of people undergoing PCIs [32]. The implementation of these guidelines is pending. Other clinical interventions arising from this study are being refined for pilot testing in the near future.

### 4.9. Phases 7, 8, and 9: Evaluation

The completion of the implementation phase signals the transition into the Model's evaluation phase. Process evaluation enables the change process by which the intervention is being implemented to be evaluated. During Phase 8 the “impact evaluation” measures the program effectiveness in terms of the intermediate objectives and changes in the predisposing, enabling, and reinforcing factors. Outcome evaluation is the final evaluation phase of the PRECEDE-PROCEED Model (Phase 9) and measures the overall program goal. Evaluation data from the two projects is reported elsewhere and are beyond the scope of this paper.

### 4.10. Summary

These case studies demonstrate the applicability of the PRECEDE-PROCEED Model within two discrete population groups. In both settings, unmet needs could have been addressed through implementing existing knowledge; however, applying the Model enabled a focused approach to intervention development that considered a range of relevant factors. As narrowing the evidence-practice gap continues to be a major health reform challenge, it was critical for both projects to take into consideration the obstacles to change for each population in developing effective interventions [36]. As interventions that consider predisposing, enabling, and reinforcing factors have the most success in implementing best practice focusing on these determinants is critical to developing successful interventions, as was demonstrated in these case studies [24, 25]. In the R-PAC Project, focussing the intervention on increasing the competencies of aged care personnel was identified as likely to have the greatest impact on delivery of an evidence-based palliative approach to older people in aged care, while the APRICA 2 Project focussed on improving the outcomes for people undergoing PCI by improving postdischarge care and increasing access to CVD secondary prevention initiatives. This systematic approach to health planning allowed for the setting of priorities and guided the focus of the interventions whilst assisting with delineation of responsibility of those professionals and organisations involved in the process [10]. Applying the Model also ensured that a realistic and applicable evaluation framework was simultaneously developed.

## 5. Conclusion

As demand for health care resources continues to increase, there is a need to ensure the systematic and critical analysis of priorities and presumed causes is undertaken [3]. The PRECEDE-PROCEED Model takes into account the multiple factors that shape health status and assists health care planners and clinicians to develop programs that intervene on factors that are both important and changeable and encourages participatory research and practice [3, 37]. A key attribute of the Model is its focus on determining the desired outcomes at the outset of the planning process [10]. Applying the Model ensured that all of the relevant environmental and nonbehavioural factors that can act as barriers to health care innovations and practice change were considered, which is an important consideration as they are often overlooked during health intervention planning [10]. 

A needs assessment is an inexact science with several factors limiting its effectiveness [4, 8, 38]. The PRECEDE-PROCEED Model addresses many of these limitations. This model demands that an inclusive process as opposed to tokenism is utilised, which ensures the active involvement of local communities and consumers in identifying, prioritizing, and responding to these needs [4]. As such, the Model prevents the needs assessment process from being ritualistic and self-justifying, by ensuring that the process is focused on facilitating health care reform [8, 38]. Although, undertaking a needs assessment implies that a change is required, there is little evidence that documenting “need” alone actually leads to effective health system change [9]. In spite of a commitment, many health services have limited capacity to reorientate health priorities and funds into new programs without engaging in a range of far-reaching reforms [6]. In these circumstances, conducting a needs assessment without a commitment to implementing recommended solutions is a lost opportunity to address identified unmet need, resolve issues, and an unnecessary and wasteful strain on scarce resources [6]. The PRECEDE-PROCEED Model challenges health services to change practices and prevents reinforcing a potentially dysfunctional status quo in service or program delivery [4, 8]. This diagnostic method increases the utility of a needs assessment in the real world setting by providing a framework which encourages identification and consideration of the environmental, social, and behavioural factors that may impact on any planned intervention. It enhances the acceptability of interventions by enabling health professionals to develop improvements that act on factors that are not only important but also amenable to the change. These case studies demonstrate the relevance of this multidimensional planning Model in targeting health care improvement strategies in dynamic environments.
